# Mechanical properties and residual stresses in multi pass AISI 304 to AISI 1018 steel welds by SMAW and GTAW

**DOI:** 10.1038/s41598-025-06063-0

**Published:** 2025-07-25

**Authors:** Shridhar, Prabhat Kumar Janapareddy, G B Veeresh Kumar, Santosh Kumar Sahu, Ali El-Rayyes, Jayant Giri, Balkeshwar Singh

**Affiliations:** 1 Department of Mechanical Engineering, National Institute of Technology - Andhra Pradesh, Tadepalligudem, Andhra Pradesh State India; 2 School of Mechanical Engineering, VIT-AP University, Besides A.P. Secretariat, Amaravati, 522237 Andhra Pradesh India; 3 Center for Scientific Research and Entrepreneurship, Northern Border University, Arar, 73213 Saudi Arabia; 4 Chemistry Department, College of Science, Northern Border University, Arar, Saudi Arabia; 5 Department of Mechanical Engineering, Yeshwantrao Chavan College of Engineering, Nagpur, India; 6 Division of Research and Development, Lovely Professional University, Phagwara, India; 7 Centre for Research Impact & Outcome, Chitkara University Institute of Engineering and Technology, Chitkara University, Rajpura, 140401 Punjab India; 8 Department of Mechanical Engineering, Program of Manufacturing Engineering, Adama Science and Technology University, Kebele-14, Adama City, 1888 Ethiopia

**Keywords:** Dissimilar welding, SMAW, GTAW, Heat treatment, Microstructure, Residual stresses, Mechanical properties, Mechanical engineering, Characterization and analytical techniques

## Abstract

Welding is a highly reliable, efficient, and widely used technique for joining metallic structures. In this study, AISI 1018 Mild Steel (MS) and Stainless Steel (SS) AISI 304 were joined using Shielded Metal Arc Welding (SMAW) and Gas Tungsten Arc Welding (GTAW) multipass welding methods. Post-Weld Heat Treatment (PWHT) was performed on the weldments using an induction furnace to analyse the effects of PWHT on microstructural variations, mechanical characteristics and residual stresses of dissimilar weld joints. The results revealed that PWHT improved the mechanical properties of the weld and substantially reduced residual stresses by ~ 48.5% in both SMAW and GTAW weld regions. Additionally, PWHT enhanced impact toughness, with increases of 17.3% for SMAW weldments and 21.4% for GTAW weldments. The weld area exhibited the highest hardness values across all weldments; however, a slight reduction in tensile strength was observed after PWHT due to material softening effects.

## Introduction

Many discrete parts are often joined to create more significant, complex components, with welding standing out as a highly reliable and prominent technique for assembling metallic structures. Welding involves achieving localised coalescence through applying heat, pressure, or integration of both^1^. The primary goal of the welding industry is to produce high-quality products that are lightweight, cost-effective, and efficient. To achieve this, manufacturers are increasingly focusing on dissimilar metallic joints, which offer the potential to reduce weight and production costs while maintaining structural integrity. There is a wide range of usage of dissimilar metal joints in nuclear plants, the automobile industry, electronics, marine, chemical industries, pressure vessels, etc. Different materials welded joints make it conceivable that the right materials intended for parts or construction are exposed to several stresses at various locations^2^. Welding dissimilar materials is a highly intricate and demanding process. The challenges are further compounded by the formation of intermetallic phases, potentially causing increased brittleness and complications in the Heat-Affected Zone (HAZ). Variations in melting temperatures, dilution rates, thermal expansion coefficients, and thermal conductivity are key factors that often result in defects during dissimilar welding^3^. Selecting the most appropriate welding technique is essential to ensure a strong and cost-effective joint.

The high corrosion resistance is a key feature of austenitic Stainless Steel (SS), given that they also provide good ductility, strength, toughness, and weldability, which makes them an essential engineering material^4^. Among these steels, AISI 304 grade SS is commonly used in structural applications in the nuclear industry, such as reactor coolant pipes, chemical & petrochemical sector, valve bodies, vessel internals, and others. AISI 304 is extensively utilised across various sectors, with one of its most prominent applications being in the storage and transportation of Liquefied Natural Gas (LNG)^5^. Additionally, due to its exceptional properties, this grade is widely employed in cryogenic vessels, screws, valves, springs, and equipment for industries such as fertilisers, petrochemicals, and chemicals^6^.

Due to its cost-effectiveness in manufacturing, widespread availability, excellent mechanical properties, and high performance, MS AISI 1018 is widely used in numerous engineering fields, including power plants, submarines, pipelines, and automobiles. By combining dissimilar metals such as SS and MS, the costly austenitic SS, known for its superior corrosion resistance, can be utilised in high-temperature zones. At the same time, the more economical mild carbon steel can be applied in lower-temperature areas. Such dissimilar metal welds are designed to operate at elevated temperatures for extended periods during power generation and are widely adopted across various industries. However, weld failures can occur during welding and service due to factors such as phase differences, transitions, the formation of brittle phases, carbide precipitation, residual stresses, and other related issues^7^. Dissimilar welds are also commonly used in marine structures, such as boat frames and fittings, where SS components are joined to MS hulls or decks. SS tanks and vessels used in brewing and distilling processes are often connected to MS supports through welding. Similarly, dissimilar welds are employed in offshore platforms and marine environments to connect stainless steel components (e.g., handrails, access ladders) to mild steel structural elements.

Thick sections are often joined using multiple passes, depending on the thickness of the workpiece. This multipass welding technique is commonly used for repairs, surface hardfacing, and similar applications. However, changes in mechanical properties and microstructure can occur due to varying thermal cycles during welding. The localised heating and rapid cooling inherent in the welding process and differences in material solidification times often lead to the generation of residual stresses in welded structures^8^.

Welding, casting, machining, surface coatings, heat treatment, and similar processes often form residual stresses, which often exist as either tensile or compressive. Tensile residual stresses may lead to stress corrosion cracking and, in certain conditions, may cause brittle fractures. Conversely, compressive residual stresses can enhance buckling strength when the material is subjected to compressive loads. Residual stresses significantly influence the fatigue behaviour of weldments and can lead to sudden, catastrophic failures of structural components. However, compressive residual stresses are beneficial as they reduce mean and maximum stresses, thereby extending the fatigue life of welded parts^9^. Residual stresses significantly impact the ultimate strength and longevity of welded structures by influencing distortions, fatigue performance, and the initiation and propagation of fractures. It has been well-documented in the steel industry, particularly in shipbuilding, that residual stresses caused by welding reduce the ultimate compressive strength of structures^10^.

Residual stresses can be measured using various non-destructive, semi-destructive, and destructive techniques. Semi-destructive and dangerous methods, which are mechanical, involve calculating the initial stress state by evaluating the displacement caused by stress relief when material is removed. Non-destructive evaluation methods, including Barkhausen noise analysis, neutron diffraction, X-ray diffraction (XRD), and ultrasonic techniques, provide alternative residual stress assessment methods without damaging the component^11^. The need for PWHT to reduce welding-induced residual stresses depends on material thickness and properties. However, in particular critical service environments, residual stress mitigation in both the weld metal and HAZ becomes essential regardless of material thickness, as these stresses may significantly affect structural integrity and performance.

Sun et al.^12^ employed a thermal-metallurgical-mechanical model to analyse residual stress and hardness variations with dilution in multipass GTAW and SAW of SA508 steel. Their results showed good agreement between experiments and simulations, revealing increased weld hardness and reduced residual stresses in the weld zone and HAZ at higher dilution levels. Ganesh et al.^13^ compared A-TIG and MPTIG welding for SS 316LN, using FEM to predict A-TIG’s thermomechanical behavior. Ultrasonic measurements indicated lower residual stresses in A-TIG joints (225 MPa) than in MPTIG (268 MPa), due to fewer thermal cycles in A-TIG.

Giri et al.^14^ investigated weld groove design effects on residual stress distribution in thick SS 304LN pipe welds. Their FEM analysis of peak temperature distribution for each pass was complemented by blind hole drilling measurements, revealing experimental hoop and axial stresses were 20–30% higher than FEM predictions. Joseph et al.^15^ demonstrated that Inconel buttering effectively reduced residual stresses in dissimilar joints between 2.25Cr-1Mo ferritic steel and AISI 316. Musa et al.^16^ conducted FEM simulations of SMAW-processed ASTM A36 plates that showed good agreement with XRD-measured residual stresses. Saha et al.^17^ established through regression analysis that increased heat input in GMAW improved weld bead width with minimal changes in reinforcement and penetration, recommending this approach for cladding applications. Jamaludin et al.^18^ compared MS and SS fillers in GTAW of SS 304/MS joints and found superior yield and tensile strengths with SS filler. Chuaiphan et al.^19^ attributed GTAW’s enhanced impact toughness (compared to SMAW) in AISI 1020/304 joints to δ-ferrite networks within the austenitic matrix.

Residual stresses are widely acknowledged to play a critical role in the failure of joints between AISI 304 and AISI 1018 steels. However, limited research exists on the impact of PWHT on residual stresses, mechanical behaviour, and microstructural evolution in these dissimilar welds. This study presents the first comprehensive analysis of multi-pass dissimilar welding between AISI 304 and AISI 1018 using SMAW and GTAW, employing the cos-α XRD method for precise residual stress quantification. The objectives include creating multi-pass welded joints using SMAW with AST101 electrode and GTAW with ER309L filler metal, quantifying residual stresses in the as-welded condition and after PWHT, and evaluating their influence on joint performance. Additionally, the research investigates changes in mechanical properties and microstructure following PWHT through experimental characterisation. The study provides a comparative analysis of SMAW and GTAW welded joints in both as-welded and PWHT conditions, examining the effects of PWHT on microstructural evolution and mechanical properties to enhance understanding of these critical dissimilar joints.

## Materials and methods

### Materials

This study used SS AISI 304 and MS AISI 1018 with 200 × 100 × 8 mm3 dimensions as base materials. A 3.5 mm diameter AST 101 electrode was utilised in SMAW, while the ER309L filler metal with a 3.2 mm diameter was used in GTAW. The chemical compositions of the base metals AISI 304 and AISI 1018^20^ are given in Table 1, while those of the ASTM 101 electrode and the ER309L filler wire are provided in Table 2. The AST 101 welding electrode was selected for its cost-effectiveness, good mechanical properties, smooth weld transfer with minimal spatter, and self-releasing slag characteristics. Due to its compatibility and superior properties, the ER309L filler was chosen explicitly for joining the base metals in the GTAW process^21^.

**Table 1:** Chemical composition of AISI 304 and AISI 1018.

Element (wt%)	C	*P*	Si	Cr	Ni	Mn	S	Fe
AISI 304	0.056	0.027	0.27	20.01	7.95	1.13	0.006	Bal.
AISI 1018	0.15–0.20	0.040 max	--	--	--	0.60–0.90	0.050 max	Bal.

**Table 2:** Chemical composition of AST101 electrode and filler metal ER309L.

Element (wt%)	C	*P*	Si	Cr	Mn	Mo	Ni	V	Cu	S	Fe
AST101	0.15	0.028	1.06	25.23	0.54	0.11	9.65	0.07	0.05	0.015	Bal.
ER309L	0.02	0.046	0.51	24.08	1.82	0.16	13.20	--	--	0.03	Bal.

### Welding process

**Fig. 1:**
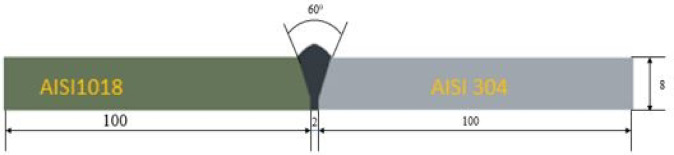
Schematic diagram of plates and V-groove.

Before welding, the plates were machined on a CNC milling machine to create a 60° single V-groove by preparing a 30° bevel on each plate, as illustrated in Fig. 1. The metals were chemically cleaned with acetone to eliminate dust, rust, and other contaminants to ensure cleanliness. Joining AISI 304 and AISI 1018 requires a precise procedure to achieve a defect-free and robust joint. SMAW was performed using an RS THYRISTOR manual welding machine, with plates tack-welded at both ends to prevent distortion. For GTAW, the DIGI-TIG PLUS 500 DP machine was employed, utilising a direct current electrode negative (DCEN) power supply with a torch. Welding was performed in an argon (Ar) environment with a constant supply rate of 10 l/min.

**Fig. 2:**
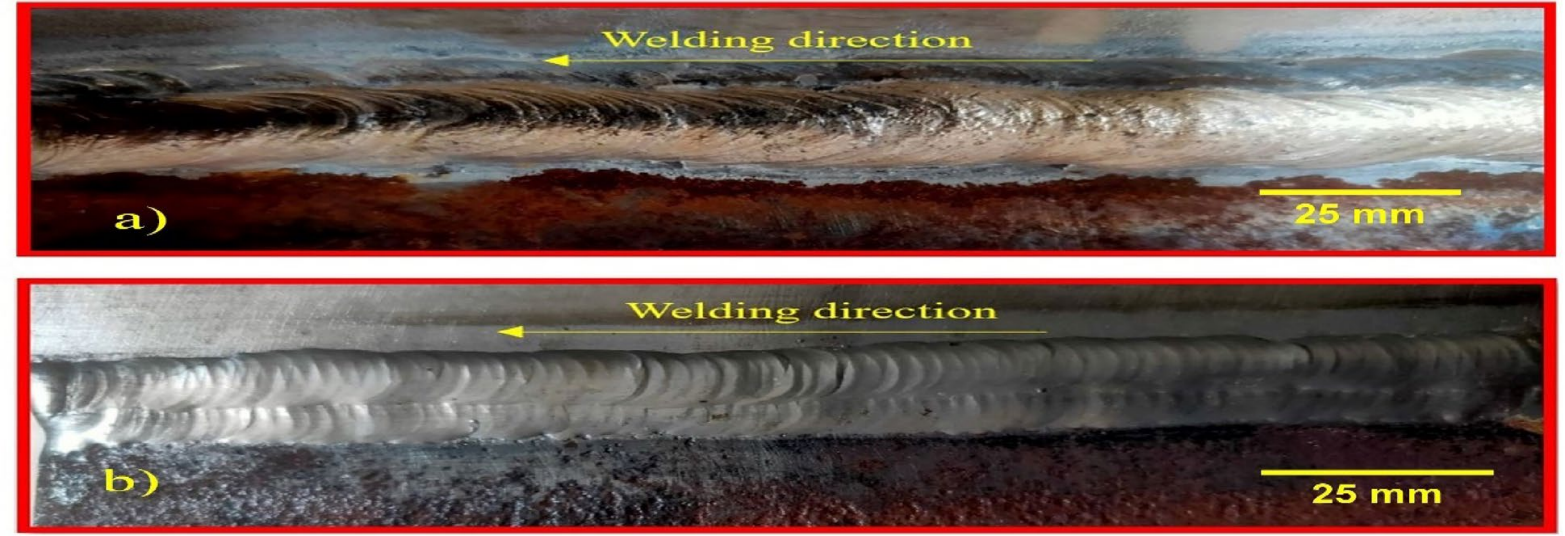
Weld bead appearance of (**a**) SMAW, and (**b**) GTAW weldments.

The welding parameters for joining these dissimilar metals, derived from existing literature and validated through bead-on-plate welding, are provided in Tables 3 and 4. The weld beads produced through SMAW and GTAW are shown in Fig. 2. To ensure the quality of the welded joints, Visual Inspection and Magnetic Particle Testing were conducted. The Magnetic Particle Testing was performed in accordance with ASTM E1444, confirming that the weldments were free of defects. For PWHT, the weldments were heated to 650 °C^22,23^ in an induction furnace, held at this temperature for 1 h, and then furnace-cooled at a rate of 100 °C per hour for 4 h before being allowed to cool to room temperature.

### Testing for mechanical properties

**Fig. 3:**
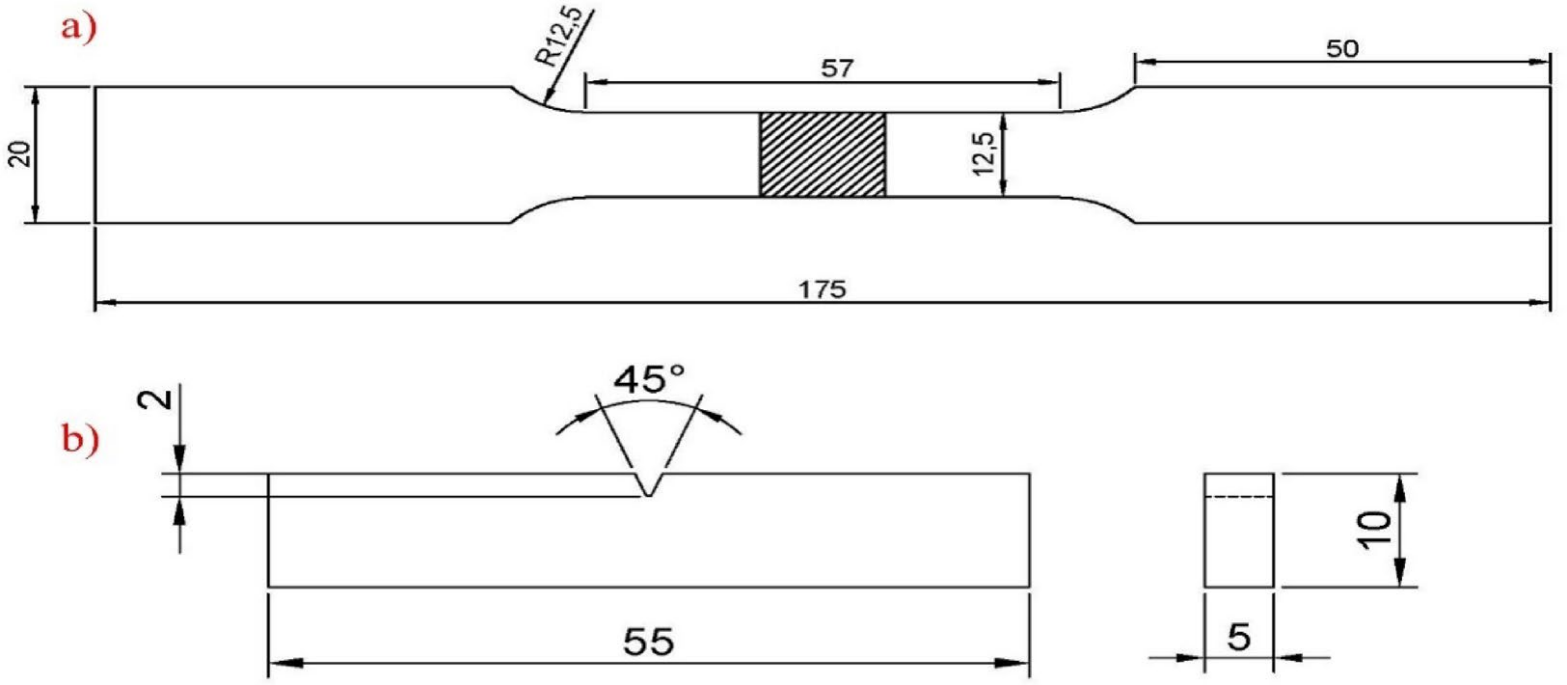
ASTM standard specimens for (**a**) tensile test and (**b**) impact test.

Various destructive tests assessed the weldments’ mechanical characteristics, including tensile, impact, and hardness tests. The tensile test used a BISS Universal Testing Machine (UTM), model no. UT-D4-0100, in accordance with the ASTM E8 standard. Specimens were prepared and tested at room temperature following the standard guidelines. Charpy V-notch specimens were used for the impact test. The test was conducted on sub-sized specimens with dimensions of 55 × 10 × 5 mm^3^ at room temperature, following the ASTM E23-12 C standard, using an FIE pendulum impact testing machine, model number IT-30. Figure 3 illustrates the schematic diagrams of the test specimens used for tensile and Charpy impact testing. The hardness test was carried out to assess the ductility of the specimens. A Rockwell hardness tester (RAB250) was used to map the hardness at various locations of the weldment, adhering to the ASTM E18-20 standard.

**Table 3:** Welding parameters for GTAW.

Pass (number)	Current (A)	Voltage (V)	Welding speed (mm/s)	Filler	Filler diameter (mm)	Shielding gas
1	130	15	1.3	ER309L	3.2	Argon (10 l/min)
2	110	15	1.12			
3	110	12	1.2			

**Table 4:** Welding parameters for SMAW.

Pass (Number)	Current (A)	Voltage (V)	Welding speed (mm/s)	Electrode	Electrode diameter (mm)
1	150	24	1.85	AST101	3.5
2	150	24	1.58		
3	130	22	1.76		

### Microstructural characterisation

Microstructure analysis of the weldments provides detailed insights into grain orientation, structure, boundaries, and irregularities that may contribute to failure. For this analysis, specimens were cut to 30 × 10 × 8 mm³ dimensions along the transverse section. Standard metallographic procedures were followed, including polishing with emery paper grades 1/0, 2/0, 3/0, and 4/0, and etching to enhance image contrast and reveal distinct microstructural features. Chemical etching was performed according to ASTM E407, using 2% Nital for the AISI 1018 side and Aqua Regia for the AISI 304 side. The microstructural images were captured using an IQM-50 microscope. Additionally, Scanning Electron Microscopy (SEM) analysis was conducted using a VEGA3 model (Tescan, Czech Republic) and Energy-Dispersive X-ray Spectroscopy (EDS) was utilised to identify the elemental composition at specific points and along lines to examine various zones of the weldments. These techniques provided a comprehensive understanding of the microstructural and compositional characteristics of the weldments.

### Residual stress measurement

The integrity of a component is significantly influenced by the nature (tensile, compressive, uniaxial, biaxial, or triaxial), magnitude, and gradients of residual stresses. Precision, resolution, and accuracy are critical factors when selecting a residual stress measurement technique. This study measured residual stresses in the weldments using a Portable X-ray Residual Stress Measurement Analyser developed by Pulsetec Industrial Co., Ltd. The specific model employed was the m-X360n, which operates at 30 kv with a target current of 1.0 ma and utilises the cosα technique.

XRD is a versatile and widely recognised non-destructive technique for residual stress analysis, with two principal methods: sin²Ψ and cosα. Unlike the traditional sin²Ψ method, the cosα technique is a relatively recent approach that allows stress determination in a single irradiation, making it more efficient. The XRD technique is based on Bragg’s Law, which relates the angle of incidence (θ), the wavelength of X-rays (λ), and the spacing between atomic planes (d) in a crystal lattice. The law is expressed as: nλ = 2dsinθ, where n is an integer representing the diffraction order. When X-rays strike a crystalline material, they scatter in an orderly manner from atomic planes, producing diffraction patterns that form concentric rings known as Debye-Scherrer rings.

**Fig. 4:**
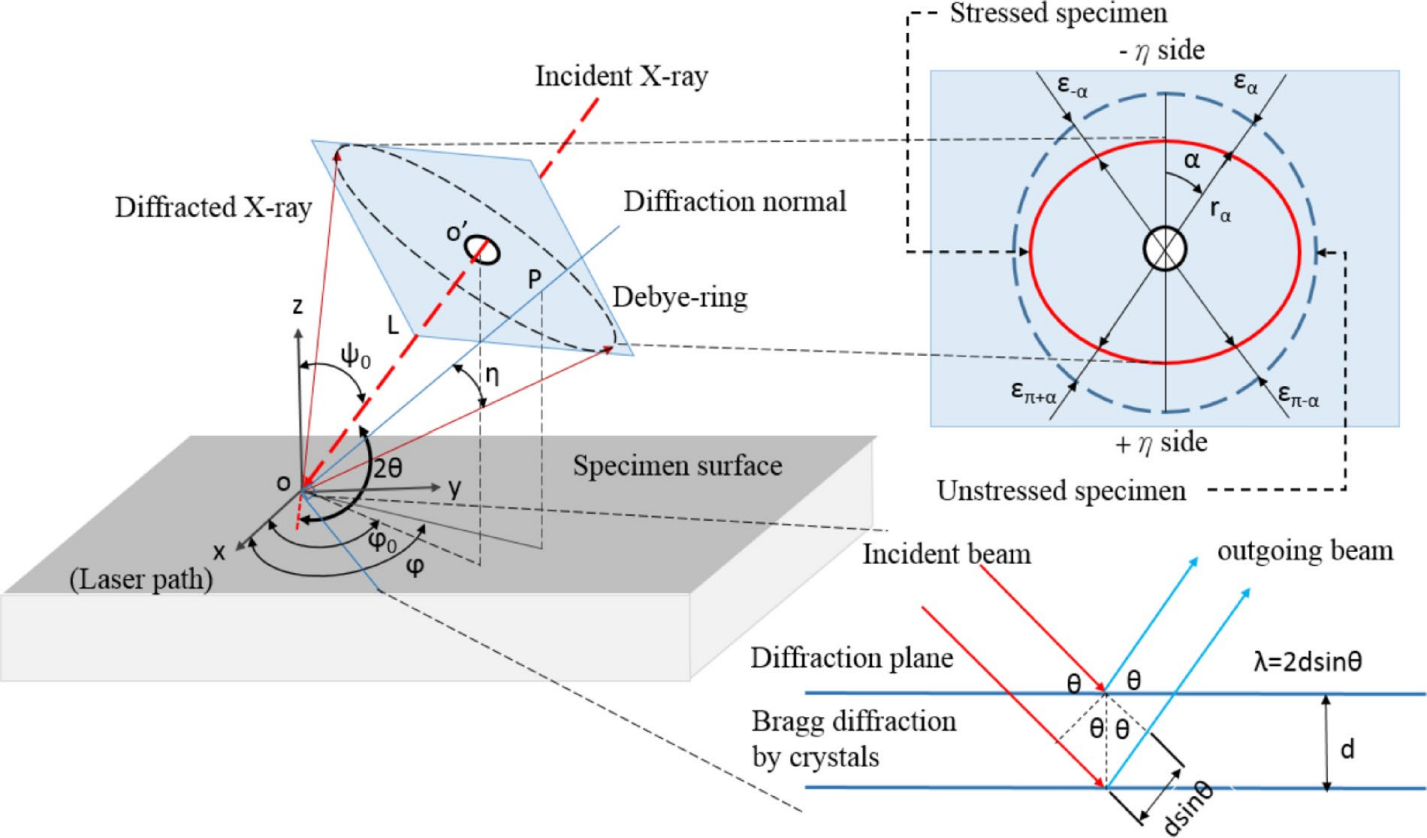
Schematic illustration of the cosα method for residual stress measurement using X-ray diffraction^24^.

Residual stress induces distortion in the Debye-Scherrer rings, as illustrated in Fig. 4, causing variations in their radius depending on the angle. The cosα method calculates residual stress using Eqs. 1 and 2. A comprehensive explanation of the cosα method for residual stress measurement is provided in the literature^24,25^.

1 $${\varepsilon _{\alpha \;\;\;}} = \;\;\;\left[ {\left( {{\varepsilon _\alpha } - {\text{ }}{\varepsilon _{\pi + \alpha }}} \right)} \right]{\text{ }} + {\text{ }}\left( {{\varepsilon _{ - \alpha }} - {\varepsilon _{\pi - \alpha }}} \right)]$$

2 $$\sigma_{X}=\:\:\:-\:\frac{E}{1+\nu\:}.\:\:\frac{1}{sin2\eta\:sin2{\psi\:}_{o}}\:(\frac{\partial\:{\epsilon\:}_{\alpha\:}}{\partial\:cos\alpha\:})$$

Where, Ψ is the angle between the incident X-ray beam and the normal to the sample surface, η is the complementary angle of the diffraction angle θ, σx denotes the longitudinal stress, α is the azimuthal angle indicating the orientation of the crystal lattice, η is the angle between the incident X-ray beam and the crystal surface, E is the Young’s modulus of the material, and φ is the rotation angle around the scattering vector. The terms εα, επ + α, ε-α, and επ-α correspond to strain components associated with lattice deformation strain at four directions (α, π + α, −α, π − α), while ν represents Poisson’s ratio.

These rings provide critical information about the material’s crystal structure and residual stress distribution. This technique compares the distorted image and the Debye-Scherrer ring, enhancing its effectiveness for stress determination. By adopting a single-exposure approach, 2d-XRD simplifies data collection and analysis, significantly reducing the time required for residual stress measurement. The combination of this advancement with compact area detectors, streamlined data processing electronics, and X-ray sources has enabled the production of portable commercial tools. These instruments allow for on-site residual stress analysis without removing the workpiece or disrupting the production process, making the method highly practical for industrial applications.

## Results and discussions

### Macrostructure of the weldments

**Fig. 5:**
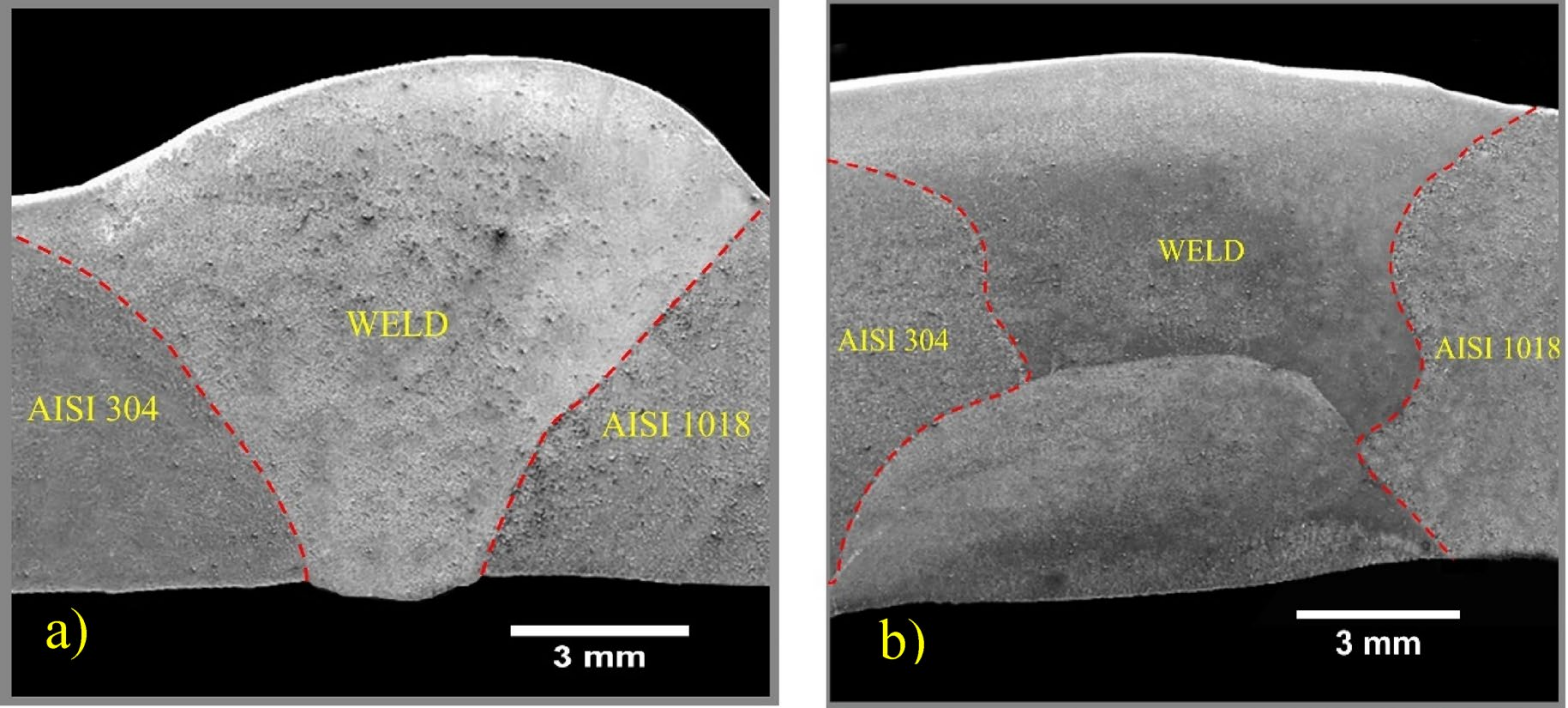
The macrostructure of SS AISI 304 weldments and AISI 1018 MS is joined by (**a**) SMAW and (**b**) GTAW.

The SMAW joint between AISI 304 and AISI 1018 was completed in three passes, and similarly, the GTAW joint also required three passes for fabrication. The cross-sectional macrostructure view of weldments fabricated by SMAW and GTAW is shown in Fig. 5. Macrographs confirmed that the joints were defect-free, with no issues such as undercut, arc strikes, or cracks due to lack of fusion. These defect-free welded joints can sustain higher loads, ensuring effective load transfer and distribution between the AISI 304 and AISI 1018 materials.

### Analysis of microstructure

**Fig. 6:**
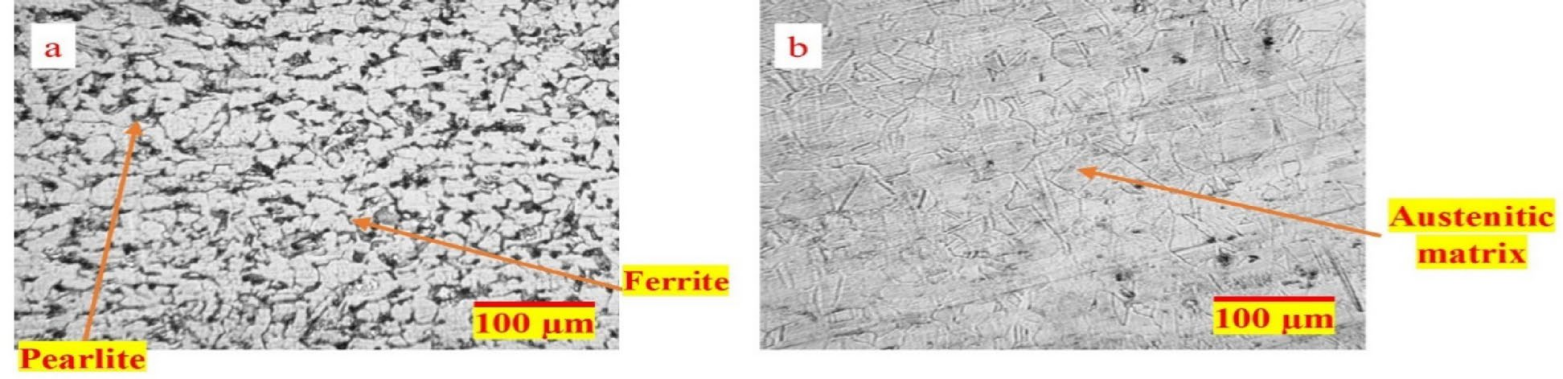
Optical images of as received (**a**) AISI 1018 and (**b**) AISI 304 base materials.

The microstructure of the base plates is illustrated in Fig. 6. The AISI 304 exhibits a distinct austenitic phase characterized by coarse, elongated grains and intermittent twinning. In contrast, the AISI 1018 microstructure is dominated by a ferrite phase (appearing as white grains) with a mixture of pearlite (dark patchy grains).

**Fig. 7:**
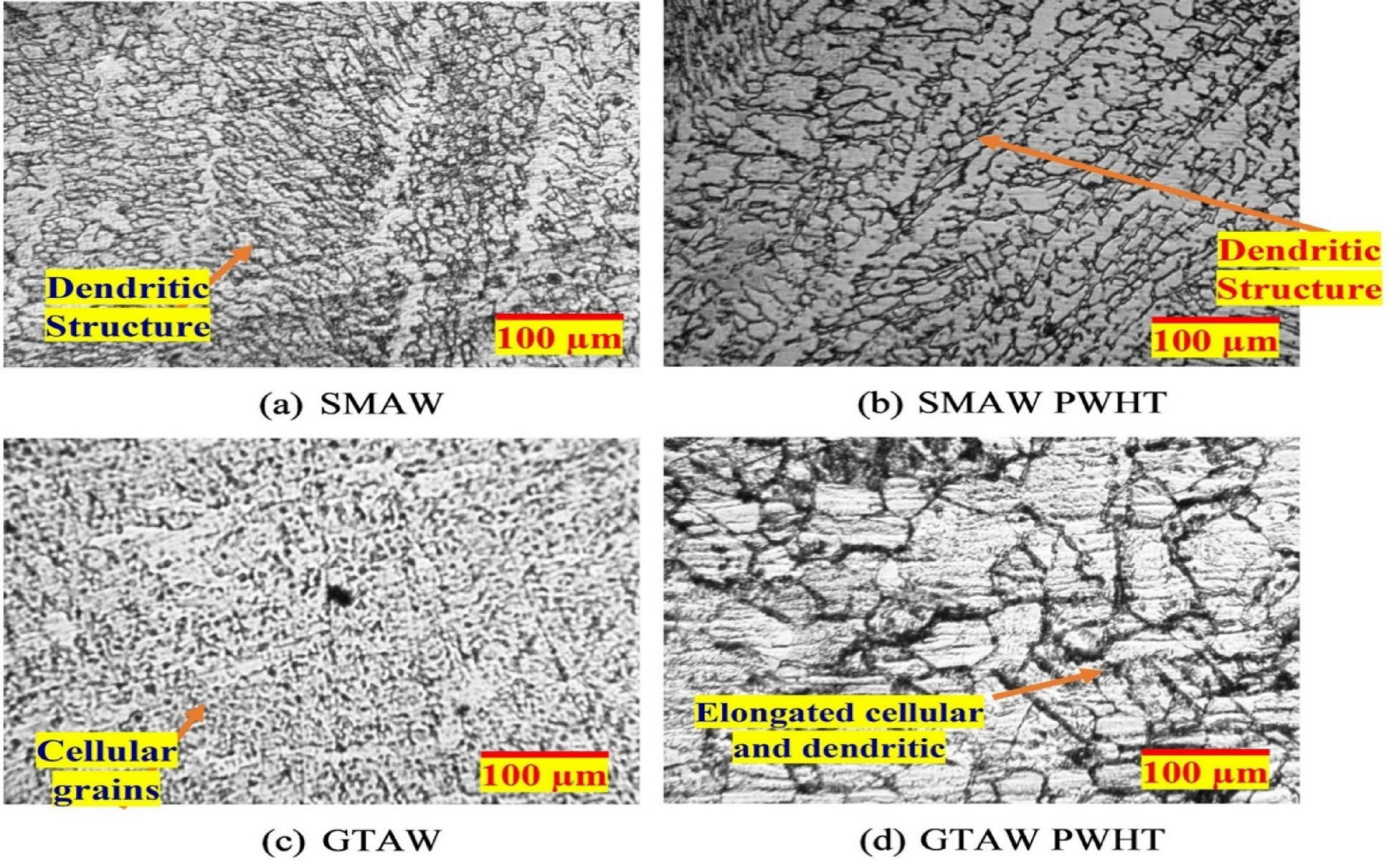
Optical images of microstructure of the weld zones.

From Fig. 7, which shows optical microscopy images of the weld zones in both as-welded and PWHT conditions, it is evident that there is no significant microstructural change. This is because the PWHT was conducted below the lower critical temperature of both SS and MS. In the weld zone of the SMAW process, a dendritic ferrite structure is observed, as seen in Figs. 7(a) and 7(b). This dendritic ferrite formation is typical in multipass welding due to variations in cooling and solidification rates as new weld passes are deposited over previously solidified weld metal. The efficient heat transfer during solidification promotes the development of this dendritic structure^26^. In contrast, the GTAW process resulted in a mixed microstructure of elongated cellular austenite and dendritic ferrite structures, as shown in Figs. 7(c) and 7(d). This variation in microstructure can be attributed to the stable arc in GTAW, which facilitates a controlled solidification process with a well-defined weld pool and solidification front. Additionally, factors such as heat input, welding speed, and micro-segregation also influence the resulting microstructure.

**Fig. 8:**
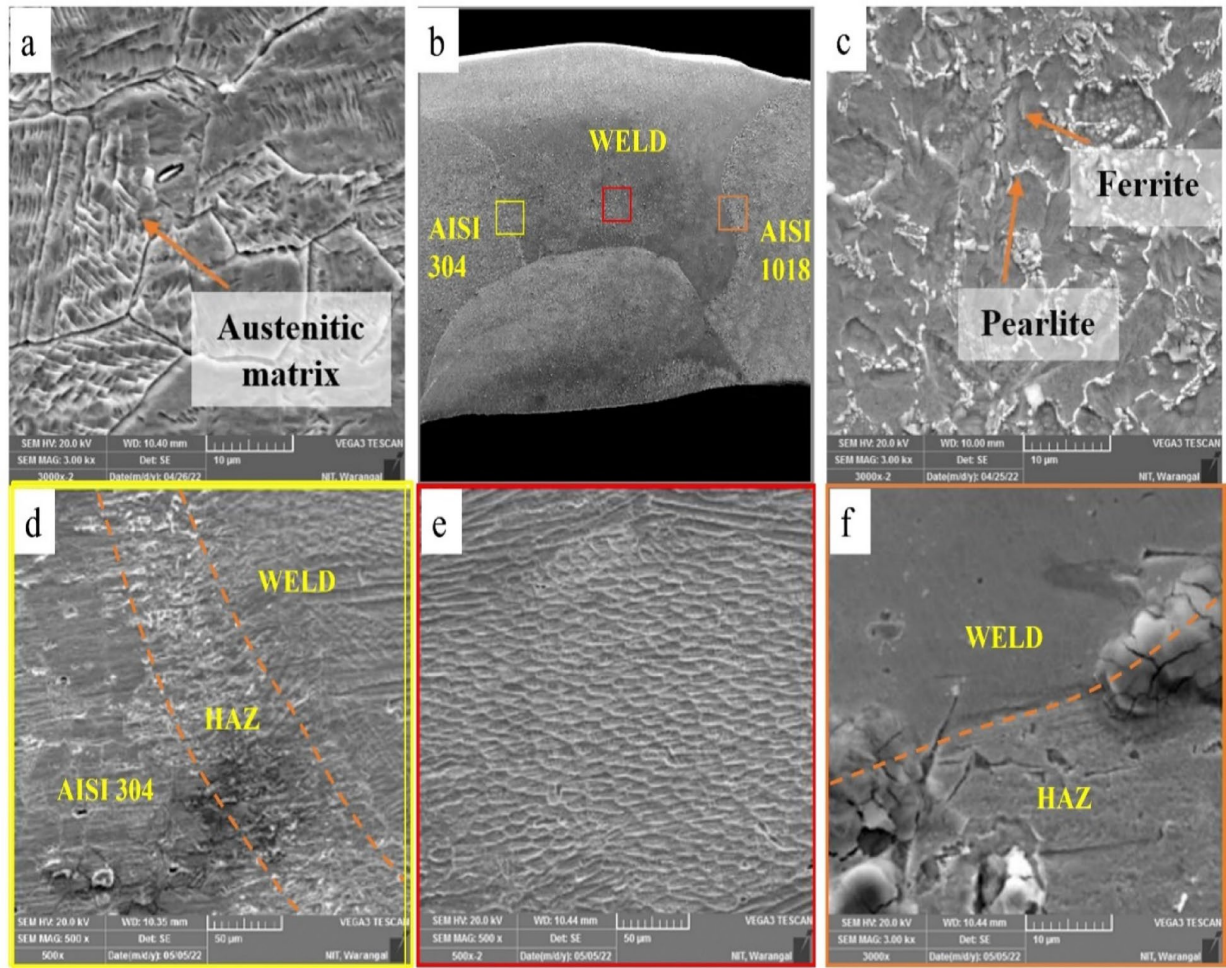
SEM images of GTAW PWHT weldment (**a**) AISI 304 base, (**b**) weld bead, (**c**) AISI1018 base, (**d**) AISI 304- weld zone interface, (**e**) weld zone, and (**f**) weld zone- AISI 1018 interface.

To gain deeper insights into the microstructural changes in the weldments after PWHT, SEM analysis was conducted on the PWHT-treated weldments. Figure 8 illustrates the various zones of the GTAW PWHT weldment, including the weld zone, AISI 304 HAZ, AISI 1018 HAZ, and base regions. Smaller grains were detected in the HAZ compared to the base material due to rapid cooling during welding and recrystallization effects induced by post-weld heat treatment, which limited grain growth and promoted grain refinement. The existence of elongated cellular and dendritic structures in the weld zone, confirmed after post-weld heat treatment, is attributed to directional solidification governed by the temperature gradient and cooling rate^23,27^.

**Fig. 9:**
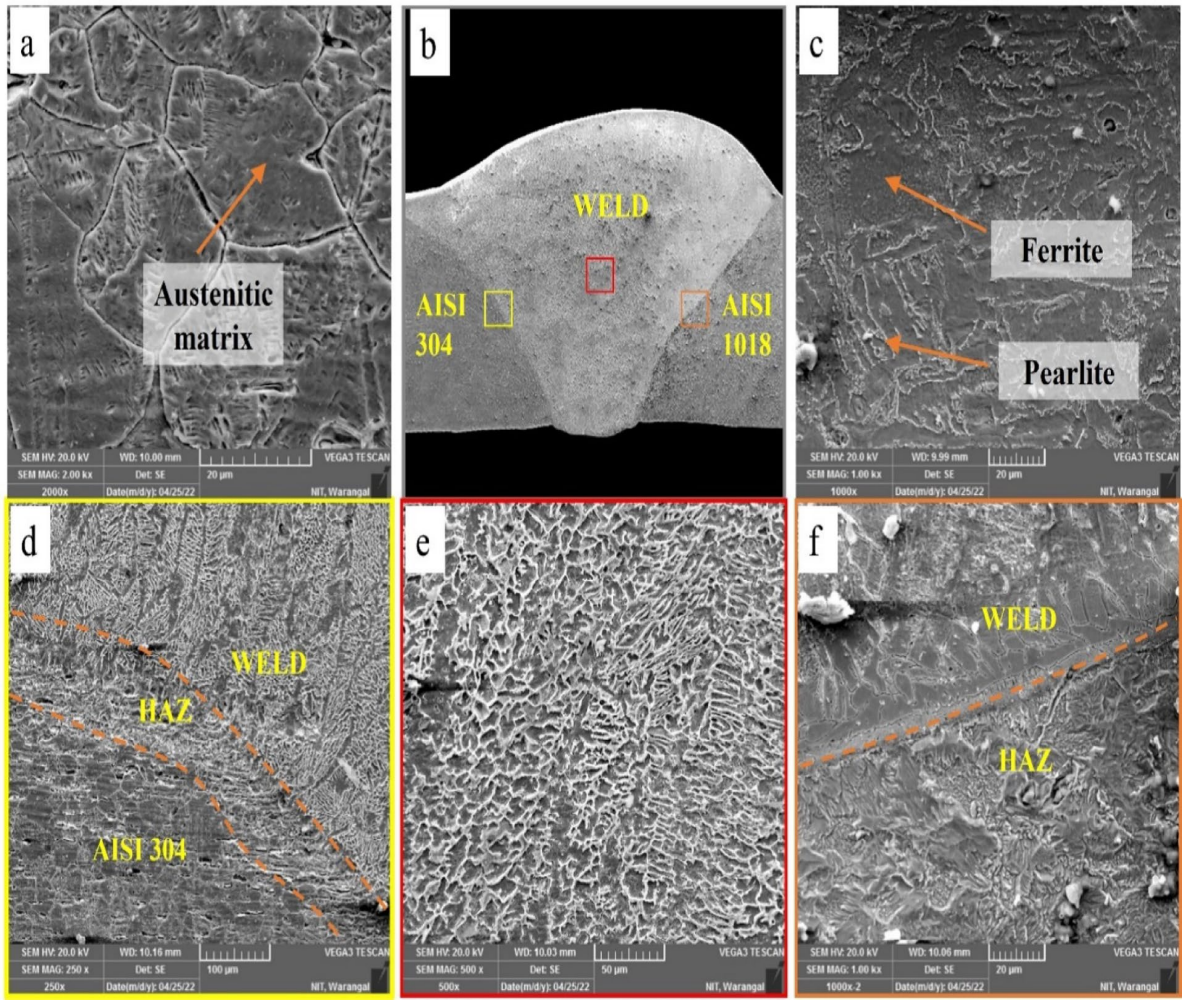
SEM images of SMAW PWHT weldment (**a**) AISI 304 base, (**b**) weld bead, (**c**) AISI1018 base, (**d**) AISI 304- weld zone interface, (**e**) weld zone, and (**f**) weld zone- AISI 1018 interface.

**Fig. 10:**
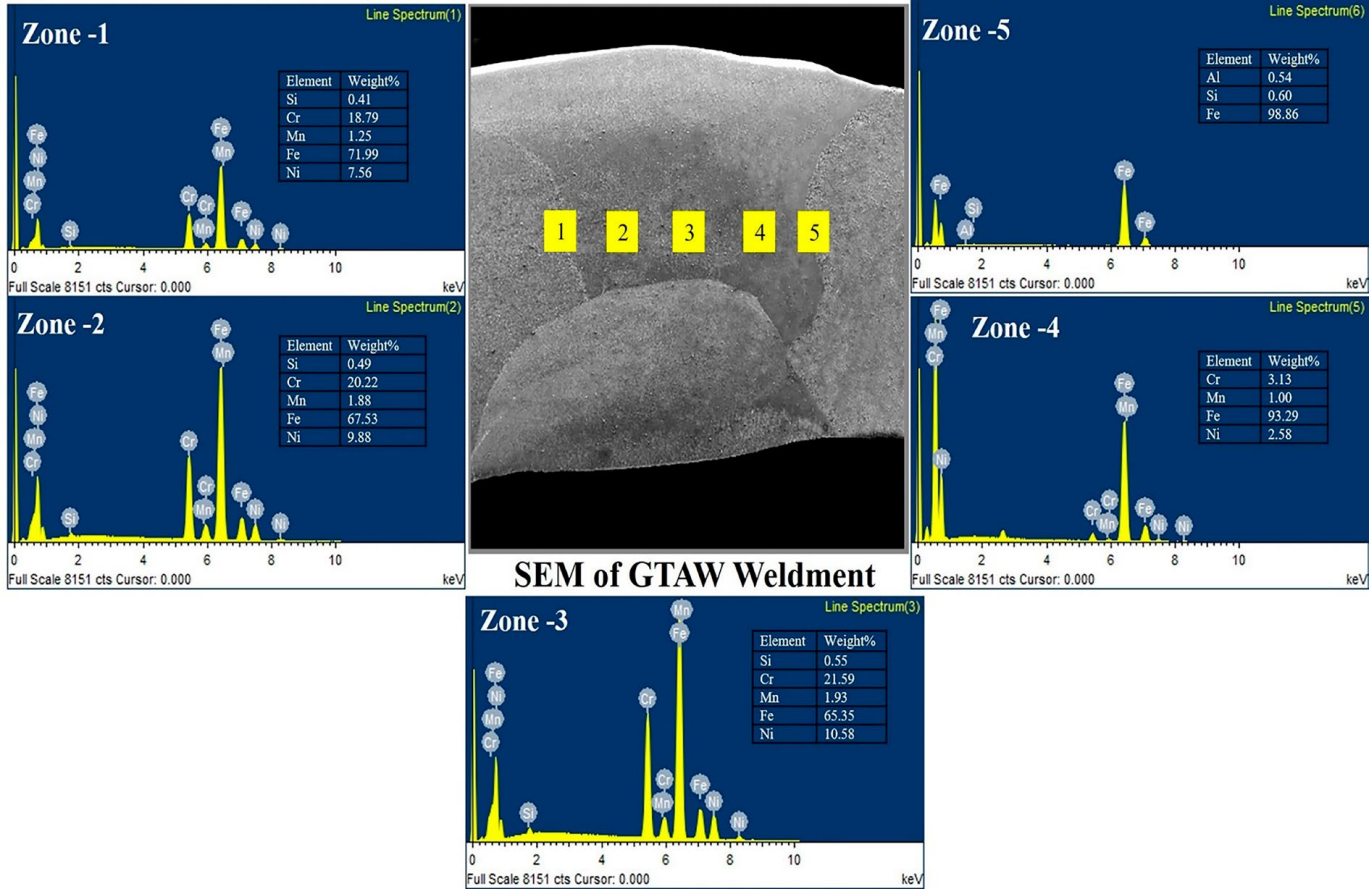
EDS profile of GTAW PWHT.

**Fig. 11:**
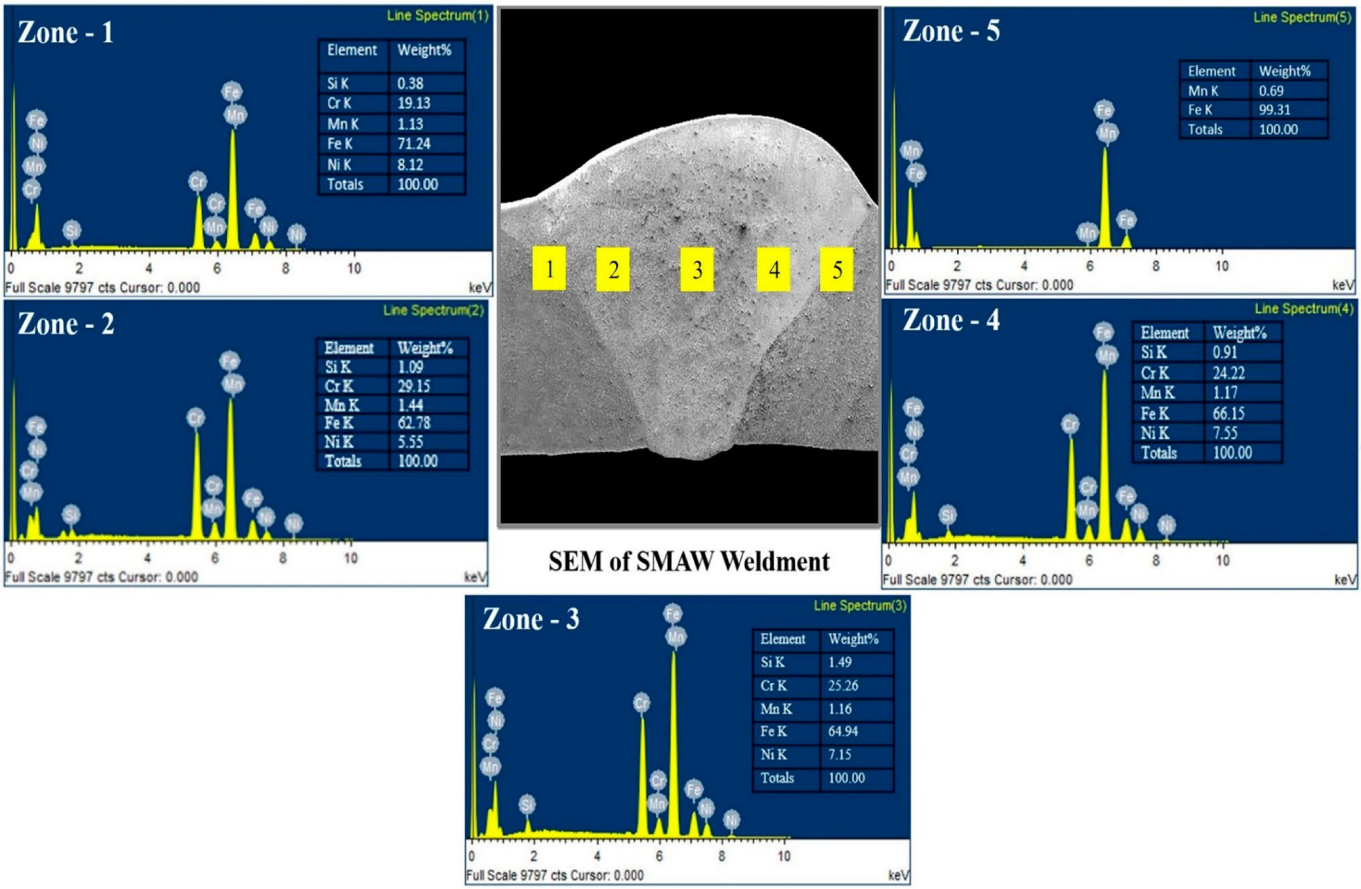
EDS profile of SMAW PWHT.

From Fig. 9, variations in grain structure are evident in the HAZ at both the AISI 304-weld interface and the AISI 1018-weld interface. Finer grains in the HAZ of the dissimilar welded joints were attributed to localized melting and fast solidification resulting from the intense thermal cycles of welding. The quick heating and cooling processes promote the formation of small grains with numerous nucleation sites. In the weld region, a distinct dendritic structure is visible, resulting from variations in cooling and solidification rates as new weld passes are deposited over previously solidified weld metal in multipass welding. In the unaffected regions, the microstructure remains similar to that of the base material. For both GTAW and SMAW microstructures, coarse grains are observed in the HAZ due to rapid solidification. This zone functions as a heat sink during welding, resulting in the development of larger grains^28^.

The EDS line spectrum of the weldments is presented in Figs. 10 and 11. The analyzed zones include: Zone-1 (HAZ of the AISI 304 side), Zone-2 (weld zone near the AISI 304 side), Zone-3 (weld zone at the center), Zone-4 (weld zone near the AISI 1018 side), and Zone-5 (HAZ of the AISI 1018 side). The EDS line mapping reveals minimal migration of elements from the filler material towards both the MS and SS sides. A higher concentration of chromium (Cr) is observed in the SMAW weldments compared to GTAW, which can be attributed to the composition of the electrode used in SMAW. The EDS line spectrum results for all weldments indicate the absence of oxide formation and no external elements beyond those specified in the chemical composition, confirming effective shielding during the welding process. In the weld zone of GTAW PWHT, the major elements detected are iron (Fe), Cr, and nickel (Ni), with minor percentages of manganese (Mn) and silicon (Si). Similarly, in the SMAW PWHT weld zone, the major elements are Fe, Cr, and Ni, with trace amounts of Mn and Si, consistent with the GTAW PWHT results.

### Mechanical properties

#### Tensile properties of the weldments

**Fig. 12:**
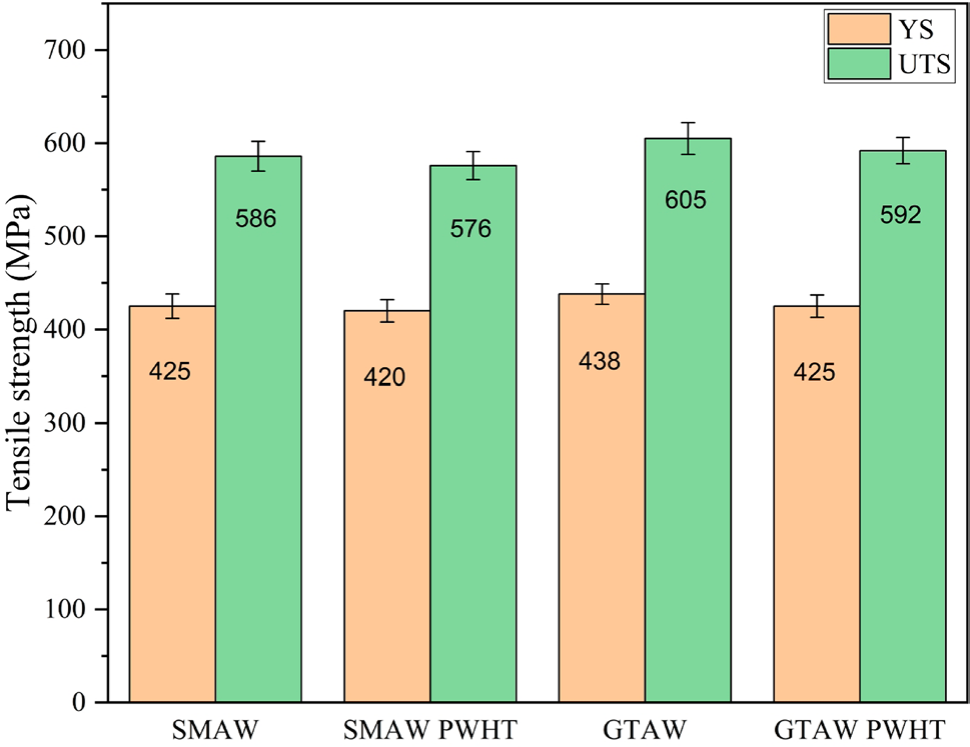
Tensile properties of the weldments of SS AISI 304 and AISI 1018 MS.

To assess the YS and Ultimate Tensile Strength (UTS) of the weldments, tensile tests were conducted. These tests provide insights into the brittleness and ductility of the welded joints. The test specimens were prepared such that the fusion zone of the weld was positioned within the gauge length. Specimens were cut along the transverse direction of the weldment in accordance with ASTM E8 standards. Tensile tests were performed at room temperature, with a strain rate of 2 mm/min. The tensile test results revealed that fracture occurred in the base material (mild steel side) for all specimens, as it is softer compared to the weld bead and AISI 304. The tensile results are summarized in Fig. 12. The UTS values for SMAW and GTAW weldments were 586 MPa and 605 MPa, respectively. After PWHT, the UTS values decreased to 547 MPa for SMAW and 574 MPa for GTAW, representing reductions of 6.66% and 5.12%, respectively. The PWHT at 650 °C resulted in a reduction of tensile strength for both GTAW and SMAW dissimilar joints between SS304 and mild steel. The slight decrement in the tensile strength is attributed to microstructural coarsening and stress relaxation effects. While GTAW welds maintained elongated cellular-dendritic structures and SMAW welds preserved their dendritic morphology, grain growth during PWHT contributed to the strength reduction. These changes were associated with decreased dislocation density and grain coarsening effects. The observed strength reduction followed typical trends for austenitic and ferritic joints subjected to PWHT^29–32^.

#### Impact test

**Fig. 13:**
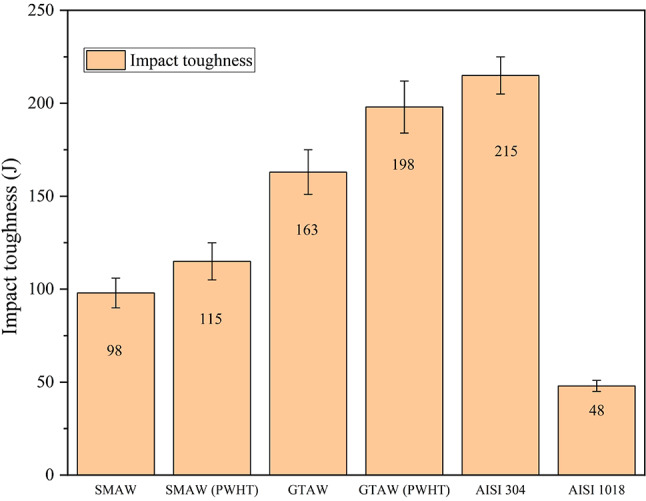
Impact toughness of weldments and base materials.

For the fabricated joints by SMAW, GTAW, and PWHT samples, the impact energy was assessed by carrying out the Charpy Impact Test according to the ASTM standard. The obtained results are presented in Fig. 13; the impact toughness of the SMA weld joint is 98 J, and the GTA welded joint is 163 J, respectively. The impact toughness of the PWHT samples, i.e. SMAW PWHT is 115 J, and GTAW PWHT is 198 J. This indicates an increase of 17.34% and 21.47% impact toughness in the SMA welded joint and GTA welded joint after PWHT, respectively. The increment of the impact energy after PWHT in both welded joints is because of the slower cooling rate and also due to the tempering and softening of the martensite. The existence of elongated cellular structures and dendritic structures in the weld zone contribute to the increased energy required for fracture at the joint, resulting in higher impact toughness for both GTAW and GTAW PWHT joints compared to their SMAW and SMAW PWHT counterparts. Studies show PWHT increases impact toughness by 15–20% in steels by homogenizing the microstructure and reducing stress concentrations. The PWHT process enhances ductility and crack resistance, ensuring better performance of welded structures under mechanical loads^23,27,29–32^.

#### Hardness test on the weldments

**Fig. 14:**
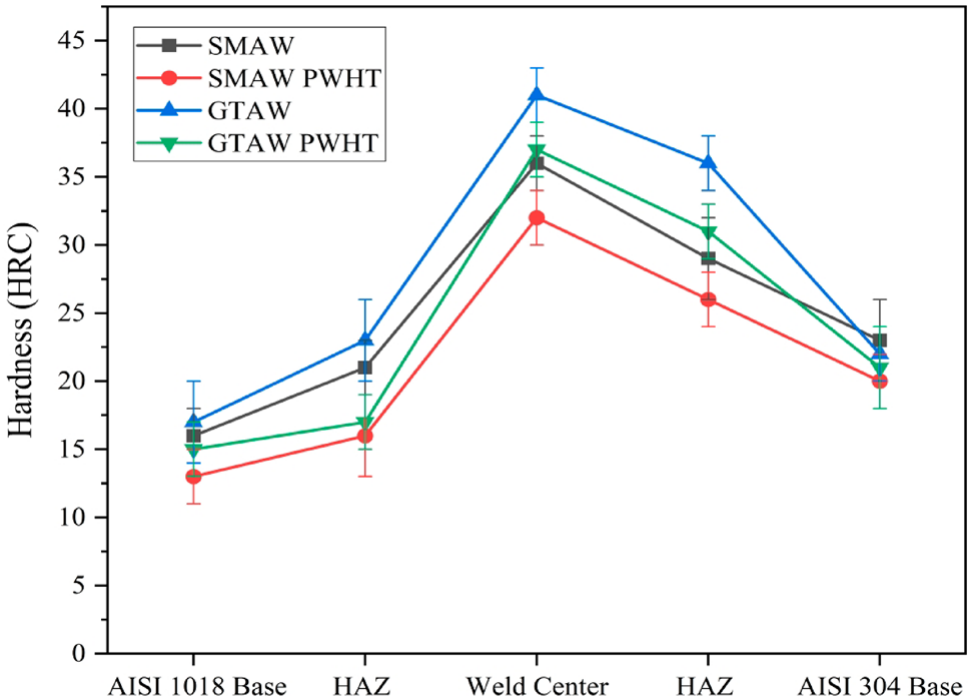
Rockwell hardness profile of weldments.

Hardness measurements were taken perpendicular to the welding direction, at intervals of 5 mm along the transverse direction and 2 mm below the top surface of the workpieces. Figure 14 illustrates the hardness profile of the weldments, showing that the maximum hardness values were consistently observed in the weld zone for all weldments. These results align with findings reported in the literature^33^. Additionally, Table 5 provides the corresponding hardness values obtained from the measurements. Compared to single-pass welding, multipass welding exhibit higher hardness values. This is attributed to the heat from subsequent passes, which delivers additional energy to the previous passes, prolonging the crystallization process. In contrast, single-pass welding lacks this subsequent heat input, resulting in a shorter crystallization process. The emergence of carbide phases further aids in the hardening of the material^34^. Among all weldments, the GTAW weldment displayed the maximum hardness value of 41 HRC in the weld zone, followed by GTAW PWHT at 37 HRC, SMAW at 36 HRC, and SMAW PWHT at 32 HRC. The higher hardness in GTAW is attributed to the greater presence of Ni in the ER309L filler metal compared to the AST101 electrode, as confirmed by EDS analysis. After PWHT, the hardness in the weld region decreased due to the disintegration of hard phases and grain coarsening^26,33^.

**Table 5:** Hardness of the weldments.

Hardness (HRC)					
	Base metal AISI1018	HAZ AISI1018	Weld center	HAZ AISI304	Base metal AISI304
SMAW	16	21	36	29	23
SMAW PWHT	13	16	32	26	20
GTAW	17	23	41	36	22
GTAW PWHT	15	17	37	31	21

### Residual stresses

Residual stress measurements were conducted in the transverse direction relative to the weld, with measurements taken at five points for each weldment, spaced 25 mm apart from the weld center. The measurements were performed using an X-ray source with Cr-Kα radiation (λ = 2.289 Å). The measurement zone was 5 mm in diameter, and a collimator with a 1 mm diameter was utilized. The ψ₀ was set to 25°, and the η was varied from 0° to 360° to capture the full Debye-Scherrer ring. For residual stress calculations, the E was taken as 210 GPa, and ν was considered as 0.28. The diffraction plane selected for SMAW weldments was {211}, while for GTAW weldments, the {311} plane was used, as these planes are optimal for the respective materials and welding processes.

**Fig. 15:**
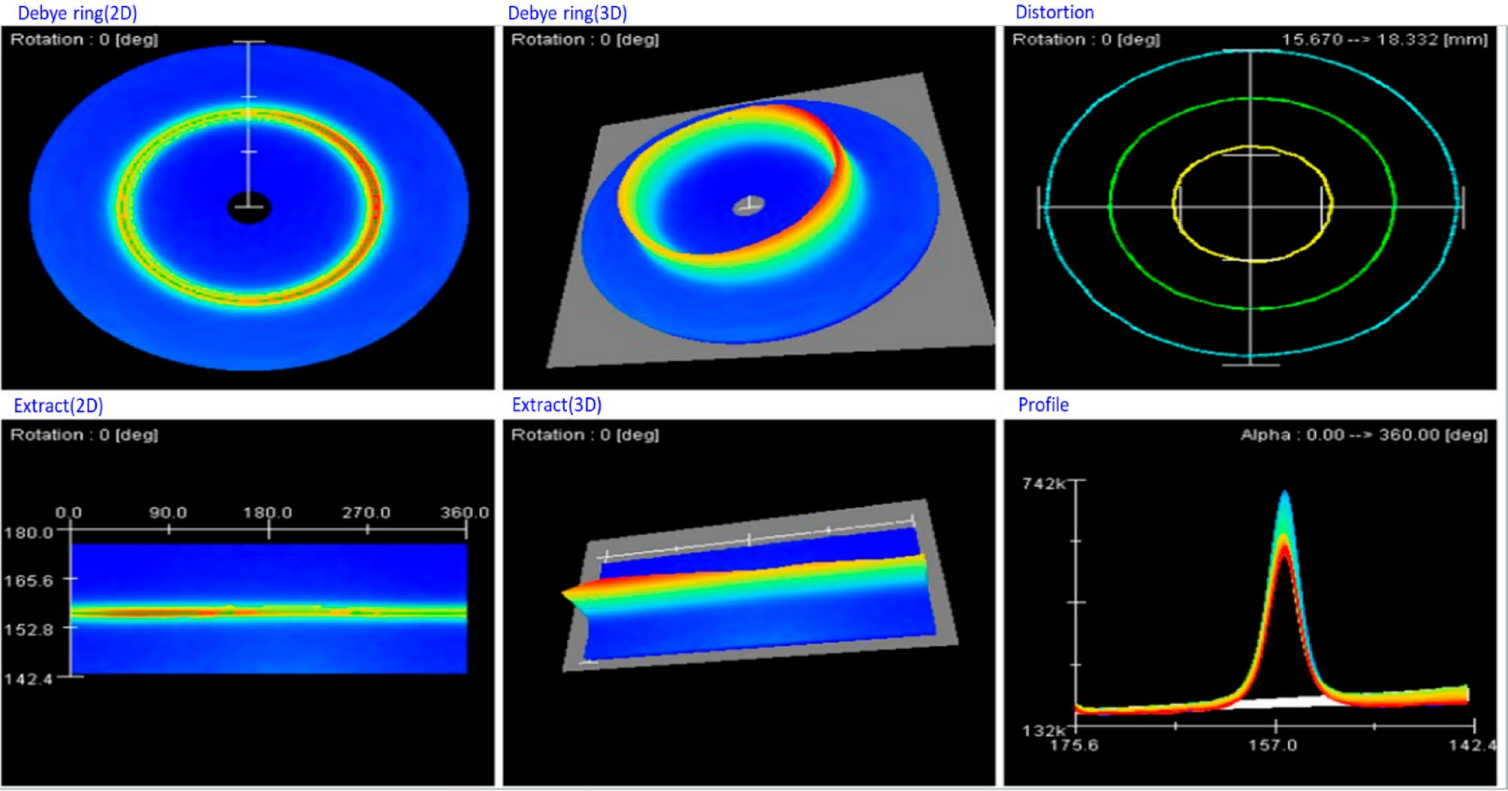
Debye–Scherrer ring information for the AISI304 base metal in a stress-free state.

**Fig. 16:**
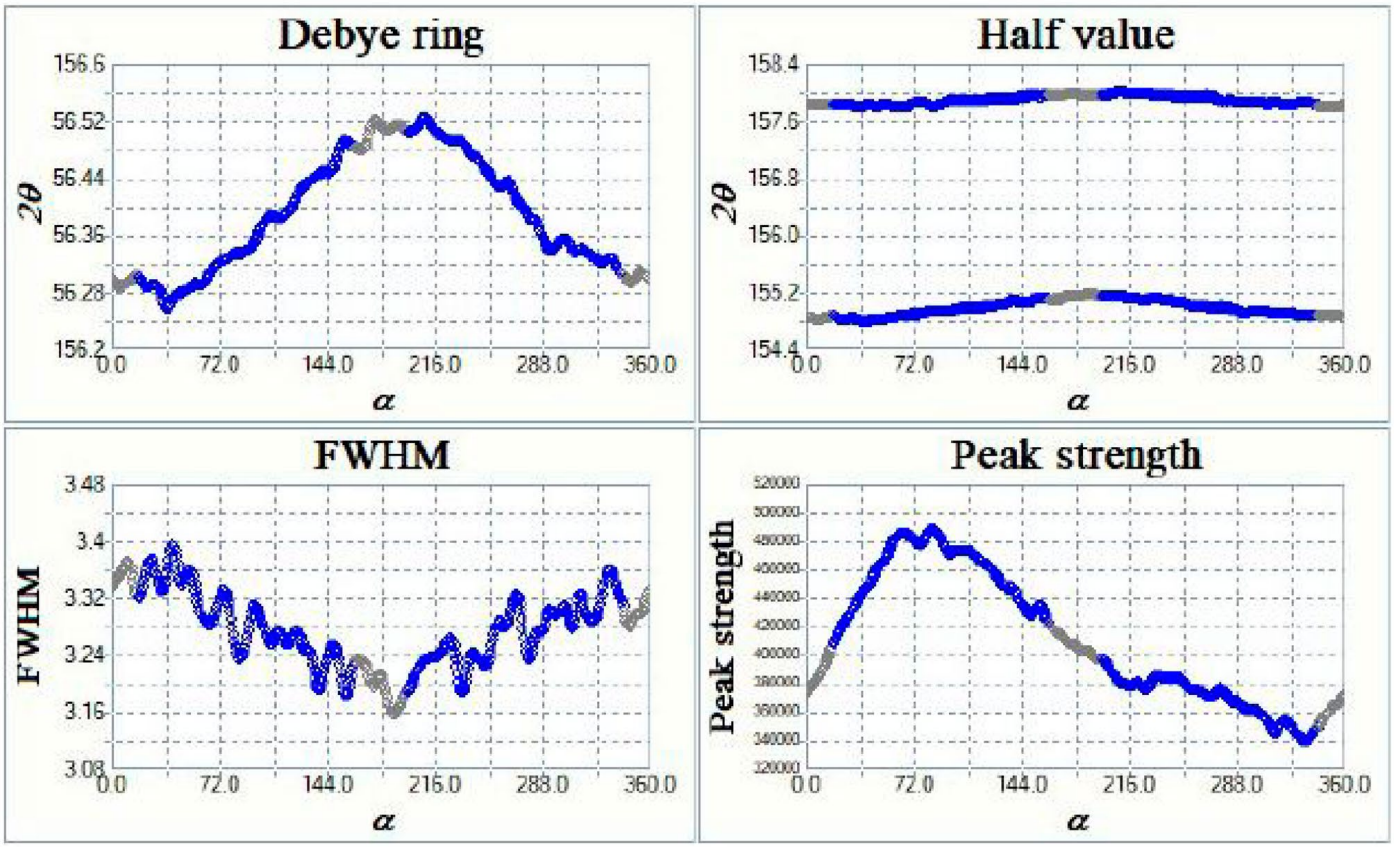
Variation of diffraction parameters as a function of α (**a**) Debye ring shift, (**b**) half value breadth position (2θ), (**c**) FWHM, and (**d**) peak intensity.

**Fig. 17:**
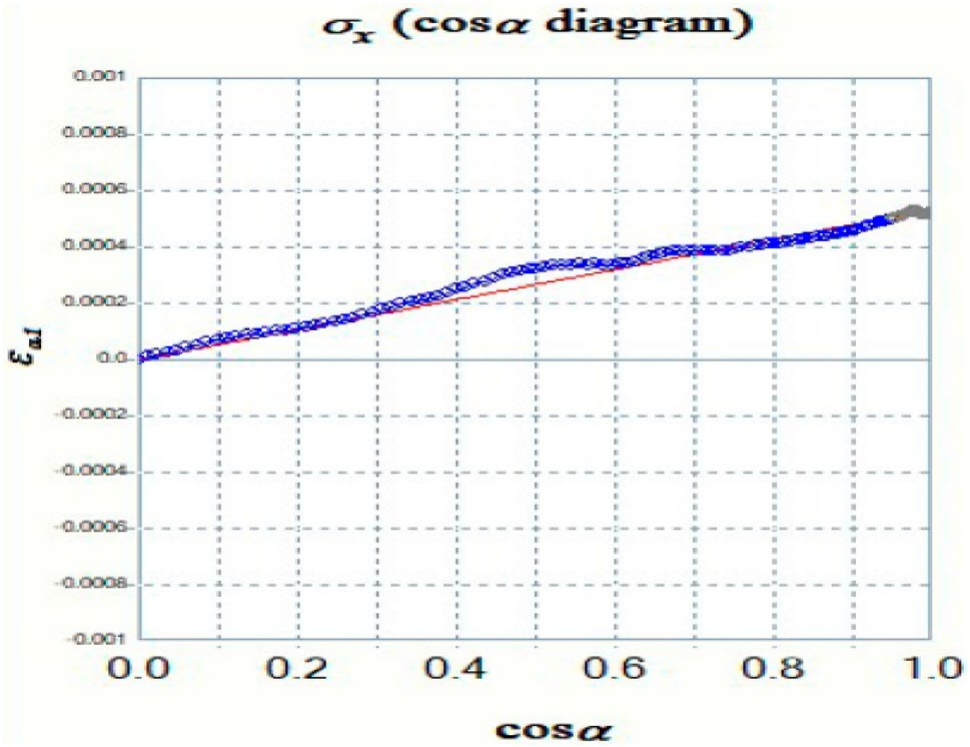
εα vs. cosα graph of GTAW weld.

The Pulstec residual stress analyser provided a comprehensive analysis of the weldments, including 3D and 2D Debye-Scherrer rings and εα vs. cosα graphs, as illustrated in Figs. 15 and 16, and 17. Figure 15 displays the Debye-Scherrer ring information for the AISI 304 base metal, showing both 2D and 3D representations of the ring, with corresponding extracted images arranged below.

The diffraction parameter variation with α-angle is shown in Fig. 16, where the complete Debye-Scherrer ring captures signals from differently oriented lattice planes. For each α-angle, corresponding φ and ψ angles represent the lattice plane orientation relative to the surface normal and stress direction. The FWHM of diffraction peaks was determined from 2θ versus intensity plots at half-maximum intensity. Figure 17 presents the εα versus cosα relationship, where the slope of the fitted line provides the residual stress value. The positive slope observed for all specimens indicates compressive residual stress. Both FWHM and peak position variations are evident from the distortion rings. These results confirm the effectiveness of the XRD cosα method for residual stress analysis in welds^35,36^.

**Table 6:** Residual stress of the weldments.

Residual stress (MPa)					
Distance (mm)	**−50**	**−25**	**0**	**25**	**50**
SMAW	45	−196	284	−225	98
SMAW PWHT	15	−50	147	−9	−3
GTAW	25	−48	224	−99	8
GTAW PWHT	10	−10	110	−20	0

The distortion of the Debye-Scherrer ring serves as an indicator of potential damage to the workpiece or the existence of excessive residual stresses^37^. A smaller FWHM in diffraction peaks reflects larger grain size and superior crystal quality, as it is closely tied to grain size^38^. Additionally, the FWHM serves as a tool for estimating the crystalline size of annealed materials^39^.

**Fig. 18:**
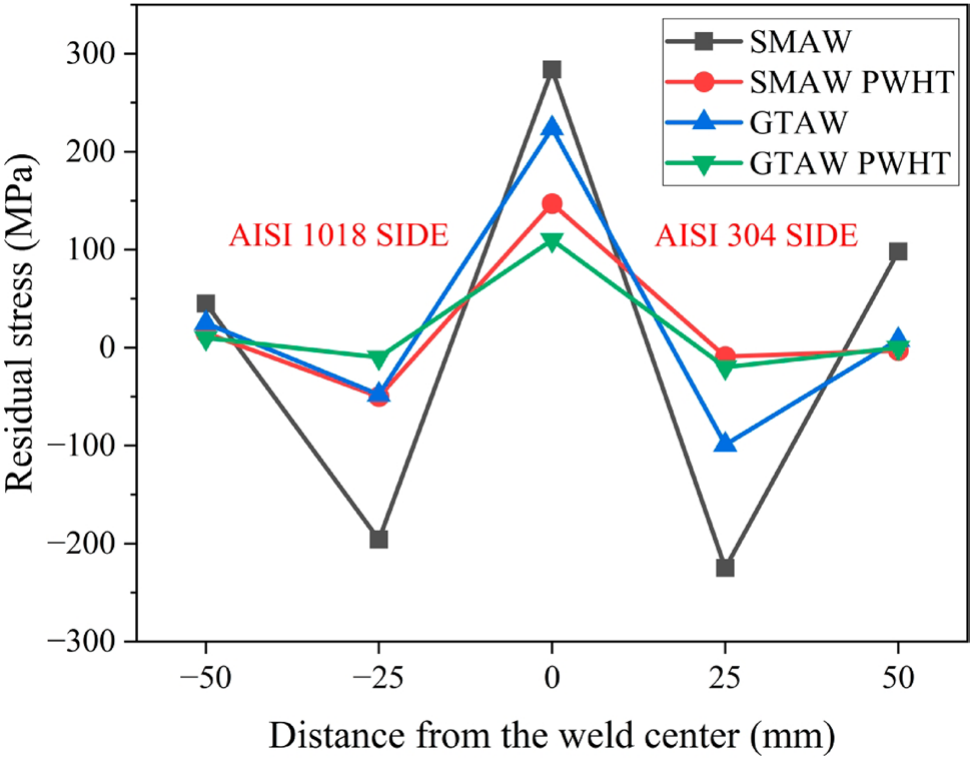
Residual stress distribution of welds before and after PWHT.

As depicted in Figs. 18, which display the residual stress distribution of weldments before and after PWHT, tensile residual stresses are focused in the weld region, and compressive residual stresses are present in areas distant from the weld. The variation in residual stresses is more significant and asymmetric in SMAW than in GTAW. The highest residual stress is located in the weld region, followed by a decreasing trend as the distance from the weld center increases, eventually stabilizing in the base material region. The residual stress distribution in dissimilar metal weldments follows an asymmetric bell curve, where the AISI 1018 side exhibits higher tensile stresses than the AISI 304 side due to its greater thermal conductivity. This asymmetry is further shaped by differences in thermal and mechanical properties, heat input, and welding parameters. The corresponding residual stress values for the weldments are provided in Table 6.

PWHT relaxes the material and redistributes stresses, leading to a reduction in tensile residual stresses around the fusion zone. The relaxation of residual stresses during PWHT is governed by factors including grain size, as the microstructure, which depends on grain size, critically influences residual stress distribution and magnitude. Generally, welds with smaller grains have more internal grain boundaries, which can result in higher residual stresses. During PWHT, the applied heat causes internal boundaries to migrate and grains to coarsen, reducing the number of internal boundaries and relaxing residual stresses.

Additionally, PWHT provides the necessary driving force to eliminate point defects, such as vacancies, and facilitates the merging and removal of dislocations. Consequently, the strain and stress fields surrounding these defects decrease, resulting in a reduction of residual stresses^40^. A comparison of residual stress distributions before and after PWHT clearly shows that PWHT effectively reduced residual stresses by ~ 48.5% in both SMAW and GTAW weldments. The 48.5% reduction in residual stress observed in this study is consistent with the findings of Huang et al.^41^ for dissimilar metal welds, though lower than the 83% reported by Paddea et al.^42^ for P91 steel, likely due to differences in phase transformation behavior. Similarly, Marques et al.^43^ emphasized that the effectiveness of stress relief is highly influenced by PWHT parameters, supporting the temperature-dependent trends observed in this study.

## Conclusions

Multipass welding of AISI 304 and AISI 1018 plates was successfully performed using SMAW and GTAW processes, followed by post-weld heat treatment (PWHT) at 650 °C in a muffle furnace to achieve effective stress relief. The key findings from the investigation on multipass dissimilar welding between these materials are summarized as follows.

- Microstructural analysis revealed a dendritic microstructure in the SMAW weldments and a mixed elongated cellular and dendritic structure in the GTAW weldments.
- The PWHT GTAW joint achieved the highest impact toughness (175 J), while the SMAW joint had the lowest (98 J). GTAW welds had the highest hardness (41 HRC), which decreased after PWHT. UTS decreased after PWHT by 6.65% (SMAW) and 5.12% (GTAW), attributed to grain coarsening and reduced dislocation density.
- Residual stresses were tensile and peaked near the weld zone, forming an asymmetric bell-shaped profile due to differing thermal conductivities. PWHT effectively reduced residual stresses by ~ 48.5% in both SMAW and GTAW weldments.

## Data Availability

All data generated or analysed during this study are included in this published article.
